# Clinical features and genotypes of six patients from four families with horizontal gaze palsy with progressive scoliosis

**DOI:** 10.3389/fped.2022.949565

**Published:** 2022-09-14

**Authors:** Lijuan Huang, Jianlin Guo, Yan Xie, Yunyu Zhou, Xiaofei Wu, Hui Li, Yun Peng, Ningdong Li

**Affiliations:** ^1^Department of Ophthalmology, The Second Affiliated Hospital of Fujian Medical University, Quanzhou, China; ^2^Department of Ophthalmology, Beijing Children’s Hospital, Capital Medical University, Beijing, China; ^3^Department of Radiology, Beijing Children’s Hospital, Capital Medical University, Beijing, China; ^4^Department of Ophthalmology, Changchun Children’s Hospital, Changchun, China; ^5^Key Laboratory of Major Diseases in Children, Ministry of Education, Beijing, China; ^6^Department of Ophthalmology, Children’s Hospital, Capital Institute of Pediatrics, Beijing, China

**Keywords:** HGPPS, *ROBO3*, scoliosis, mutation, DTI

## Abstract

**Background:**

Horizontal gaze palsy with progressive scoliosis (HGPPS) is a rare disorder mainly involved in ocular movement and spinal development. It is caused by a roundabout guidance receptor 3 (*ROBO3*) gene mutation. This study aimed to describe the clinical features of six patients with HGPPS and investigate the corresponding *ROBO3* gene mutations.

**Methods:**

Patients underwent detailed clinical and imaging examinations. Whole-exome sequencing was performed to detect nucleotide variations in the disease-causing genes of HGPPS.

**Results:**

Six pathogenic variants were detected in the *ROBO3* gene from six patients with HGPPS, including two novel compound heterozygous mutations, c.1447C > T (p.R483X) and c.2462G > C (p.R821P); c.1033G > C (p.V345L) and c.3287G > T (p.C1096F); a novel homozygous indel mutation, c.565dupC (p.R191Pfs*61); and a known missense mutation, c.416G > T (p.G139V). Patients with HGPPS had horizontal conjugated eye movement defects and scoliosis with variable degrees, as well as flattened pontine tegmentum and uncrossed corticospinal tracts on magnetic resonance imaging.

**Conclusion:**

Our genetic findings will expand the spectrum of *ROBO3* mutations and help inform future research on the molecular mechanism of HGPPS.

## Introduction

Horizontal gaze palsy with progressive scoliosis (HGPPS) is a rare disorder that involves ocular movement and spinal development (1). Patients with HGPPS lose all their horizontal conjugate eye movements, including saccades, smooth pursuit, and optokinetic responses, early in infancy but may preserve convergence and vertical eye movements. Some patients may have nystagmus and strabismus, which lead to impaired binocular vision. The onset of spinal deformity is usually insidious, presenting as progressive scoliosis in childhood or adolescence, and the pathological spine deviates laterally from the midline, which may progressively worsen over time.

Horizontal gaze palsy with progressive scoliosis is possibly inherited in an autosomal recessive manner. Mutations in the roundabout guidance receptor 3 gene (*ROBO3*) have been proven to be responsible for HGPPS (2, 3). The *ROBO3* gene is located on chromosome 11q24.2 and comprises 28 exons, and the protein encoded by *ROBO3* plays an important role in human axonal guidance. Mutations in *ROBO3* induce axonal midline crossing defects in specific populations of neurons in the hindbrain and possibly the spinal cord, leading to the absence of coordinated movement and defects in the integration of sensory information (4). To date, 61 mutations in the *ROBO3* gene have been identified in patients with HGPPS (5). In this study, we identified five novel mutations in *ROBO3* from Han Chinese patients with HGPPS.

## Materials and methods

### Patients

Six affected children (P1–6) with HGPPS were recruited in this study, four of whom were collected from two pedigrees (HP01 and HP02), and two were sporadic cases. Patients underwent physical and ocular examinations. Ocular examinations included slit-lamp examination for the anterior segment and retinoscopy for the fundus. Visual acuity was evaluated using Teller cards for younger children and Snellen charts for older children. Spherical equivalents were determined using a handheld autorefractor (Welch Allyn VS100, China) and measured in diopters. Ocular movements were evaluated in nine cardinal gaze positions. Binocular sensory status was evaluated in cooperative children using the Worth 4 dot test or Bagolini striated glasses at near and distance and by stereoacuity assessment at near using the Randot Preschool Stereoacuity test (Stereo Optical Co., Inc., Chicago, IL, United States). Multidisciplinary consultation was conducted to evaluate the nervous system and spinal development by a neurologist and orthopedist. This study was approved by the Ethics Committee of Beijing Children’s Hospital and adhered to the principles of the Declaration of Helsinki. Written informed consent was obtained from the children’s parents.

### Image acquisition

Four patients and one healthy child underwent magnetic resonance imaging (MRI) using a 3.0 Tesla system (Achieva, Philips Medical Systems, Best, Netherlands). Image acquisition was conducted using a three-dimensional (3D) T1-weighted magnetization prepared rapid gradient echo sequence covering the entire brain with the following parameters: repetition time (TR)/echo time (TE) = 8.3 ms/3.8 ms; flip angle, 12°; field of view (FOV), 180 × 180 mm^2^; acquisition matrix, 180 × 180; slice, 160; slice thickness, 1 mm; and voxel size, 1 × 1 × 1 mm^3^. Axial diffusion tensor imaging (DTI) was performed using an echo-planar imaging sequence: TR/TE = 9,300 ms/100 ms; 30 diffusion-weighted directions with a *b*-value of 1,000 s/mm^2^; and a single image with a *b*-value of 0 s/mm^2^, slice thickness of 2 mm, slice gap of 0 mm, 68 slices, acquisition matrix of 128 × 128, and an FOV of 256 × 256 mm^2^. DTI tractography was performed using a Philips IntelliSpace portal.

### Molecular diagnosis

Three milliliters of peripheral venous blood were collected from affected children and their parents. Genomic DNA was extracted using the standard phenol-chloroform extraction method. Whole exome sequencing was performed on an Illumina HiSeq X Ten platform (Illumina, San Diego, CA, United States) using the PE150 strategy. Raw reads were mapped to the Genome Reference Consortium Human Genome Build 37. Variants were analyzed using the Genome Analysis Toolkit program and searched in the dbSNP151, EXAC, gnomAD 2.1, ClinVar, and HGMD2021 databases. Pathogenicity prediction scores were obtained for missense variants using SIFT, PolyPhen-2, MutationTaster, and CADD and further confirmed by modeling the 3D structure of the protein using the PyMOL program. Sequence alignment was performed using the ClustalW algorithm in the DNAStar software package (DNAStar Inc., Madison, WI, United States). Pathogenicity assessment was based on the American College of Medical Genetics/AMP guidelines. Mutations were named following the nomenclature recommended by the Human Genomic Variation Society.

## Results

**FIGURE 1:**
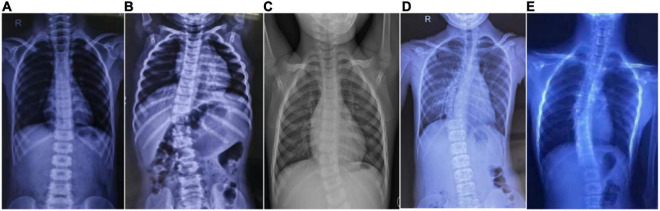
Scoliosis in five patients with HGPPS. Spine radiography images show scoliosis with variable degrees in five children with HGPPS. **(A)** A 4-year-old boy, **(B)** A 12-month-old infant, **(C)** A 4-year-old girl, **(D)** An 11-year-old girl, and **(E)** A 16-year-old boy.

**FIGURE 2:**
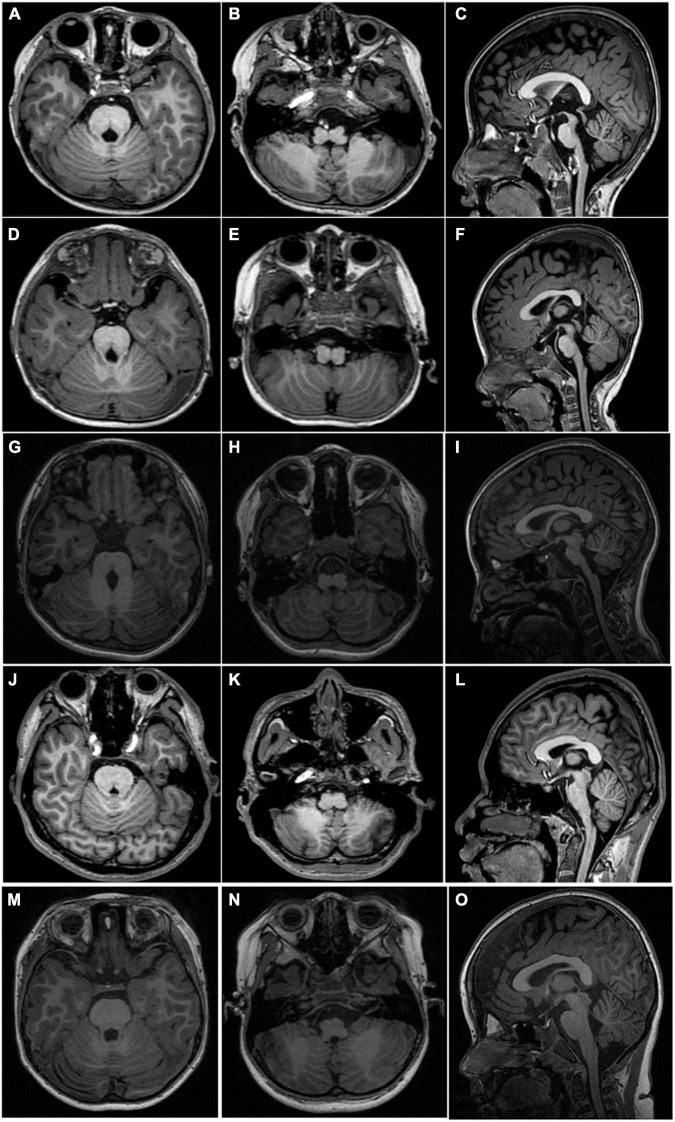
MRI scan acquired from four patients and a normal 6-year-old control. Axial T1WI shows a deep posteromedian cleft of the pons and a butterfly-like morphology of the medulla oblongata (left two columns, first four rows) in all patients. Sagittal T1WI shows flat pons and medulla oblongata and an enlarged fourth ventricle **(C,F,I,L)** in all patients. The upper panel, **(A–C)**, taken from P1; middle panel, **(D–F)**, taken from P2; **(G–I)** taken from P5; the lower panel, **(J–L)**, taken from P6. Axial T1 **(M,N)** and sagittal T1 (**O**) weighted images show normal-shaped pons and medulla of a normal 6-year-old boy.

**FIGURE 3:**
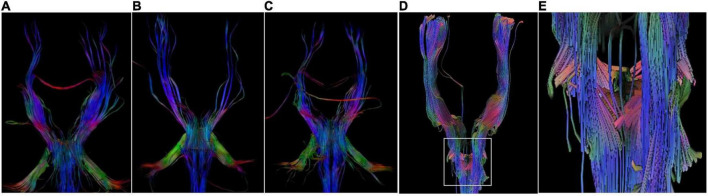
DTI Images. DTI images show a lack of corticospinal tracts decussation at the level of medulla in P1 **(A)**, P5 **(B)**, and P6 **(C)**. There are some crossed fiber bundles of bilateral corticospinal tracts in a normal 6-year-old boy **(D,E)**.

**FIGURE 4:**
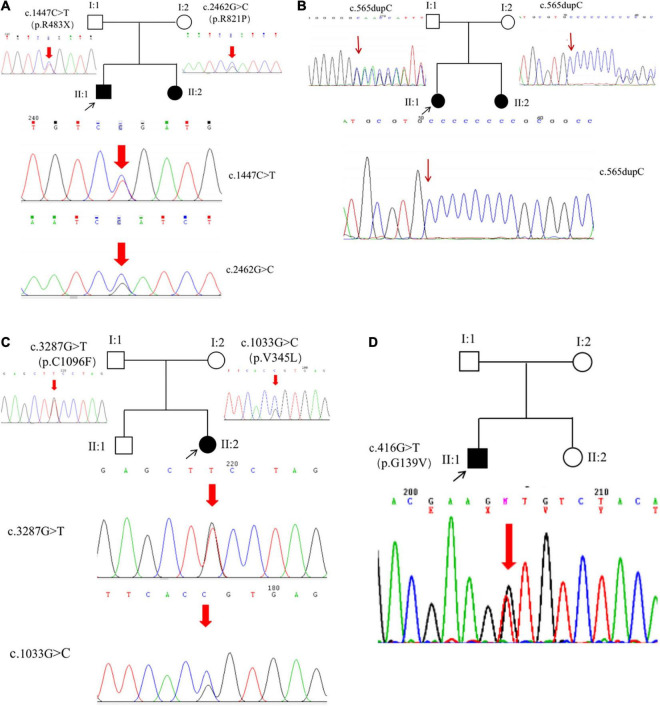
*ROBO3* mutations. Six mutations were identified from six patients, including five novel mutations **(A–C)** and a heterozygous mutation **(D)**.

**FIGURE 5:**
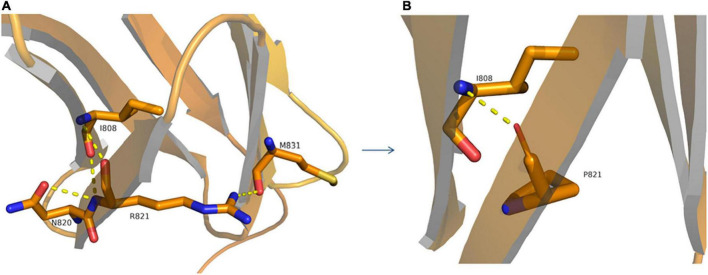
Structural model of wild-type **(A)** and p.R821P **(B)** proteins. A 3D model construction shows that the novel missense mutation of c.2462G > C (p.R821P) in the *ROBO3* gene caused largely and positively charged Arginine (R) amino acid at codon 821 to be replaced by small and uncharged Proline (P) non-polar amino acid. The wild-type arginine formed hydrogen bonds with I808, N820, and M831 **(A)**, while proline could only form a hydrogen bond with I808 **(B)**, which would damage the stability of protein structure and function.

The proband (P1) of the HP01 family was a 4-year-old boy. Medical history showed that he was referred to our hospital 3 months after birth due to the absence of horizontal conjugate eye movements. Mild nystagmus was also observed. Orthopedic consultation was conducted owing to the presence of scoliosis at 2 years of age. Radiography showed that he had mild scoliosis that deviated to his left side (Figure 1A). The proband’s sister (P2) was a 12-month-old infant with no horizontal conjugate eye movement or nystagmus around 3 months after birth. Because her brother was diagnosed with HGPPS, she was referred to our hospital for a physical examination. Although she was only 12 months old, she showed marked thoracic scoliosis convex to the right at the T12 level that developed at 8 months of age (Figure 1B). MRI revealed a deep posteromedian cleft in the pons and a butterfly-like morphology in the medulla oblongata (Figures 2A–F). DTI showed a complete ipsilateral course of the corticospinal tract without decussation at the level of the lower medulla (Figure 3A). Molecular diagnosis showed a compound heterozygous mutation of c.1447C > T (p.R483X) in exon 9 and c.2462G > C (p.R821P) in exon 16 in the *ROBO3* gene (NM_022370.3) (Figure 4A). The nonsense mutation of c.1447C > T (p.R483X) may have produced an abnormal mRNA with a premature termination codon (PTC) that would have been degraded by the nonsense-mediated decay (NMD) mechanism. The missense mutation of c.2462G > C (p.R821P) was predicted to be deleterious to protein function and structure owing to large and positively charged arginine amino acids at codon 821 being replaced by small and uncharged proline non-polar amino acids through an online analysis using SIFT, PolyPhen-2, MutationTaster, and CADD. This was confirmed by a 3D model construction using the PyMOL program (Figure 5).

The proband (P3) in pedigree HP02 came from a non-consanguineous family. The patient was a 4-year-old girl who visited our hospital as both of her eyes were unable to move outward but were able to converge inward. Ocular movement examination showed that her horizontal conjugate eye movement was completely absent, with mild nystagmus at attempted abductions. Vertical eye movements were intact. She had a best-corrected visual acuity of 8/20 in both eyes and a stereopsis acuity of 80 s of arc. Radiographic examination revealed mild scoliosis of the spine (Figure 1C). The patient was diagnosed with HGPPS based on her symptoms and signs. Moreover, her younger sister (P4) was confirmed to have HGPPS based on the absence of horizontal conjugate eye movement and molecular diagnosis at the age of 2 months. A homozygous mutation of c.565dupC (p.R191Pfs*61) in exon 3 of the *ROBO3* gene (NM_022370.3) was detected in both patients (Figure 4B). This small duplication can lead to an open reading frame shift and produce an abnormal mRNA with PTC, which can be subsequently degraded by the NMD mechanism.

**FIGURE 6:**
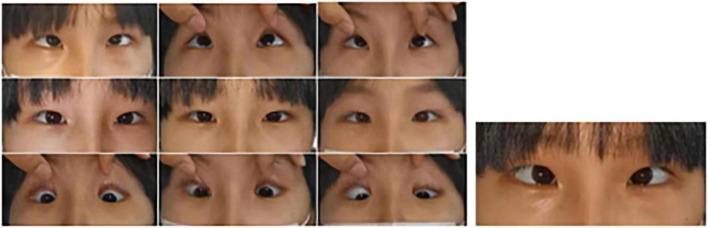
Images of eye movements of P5. The patient had a lack of conjugate horizontal movements but preserved vertical gaze and bilateral convergence.

Proband 5 (P5) was a 11-year-old girl (sporadic case) who presented with an absence of horizontal conjugate eye movements and nystagmus at the age of 3 months. Her convergence and vertical eye movements were intact (Figure 6). Her best visual acuity was 8/20 in the right eye and 10/20 in the left eye. She had good ocular alignment with a stereopsis acuity of 60 s of arc at the primary gaze position. She developed scoliosis at around 6 years of age and underwent orthotic treatment of the spine with a brace at 9 years of age. Imaging examinations showed thoracic scoliosis convex to the right on radiography, a deformed flattened pons, and a butterfly-like medulla oblongata on MRI, and a complete ipsilateral course of the corticospinal tracts without decussation on DTI (Figures 1D, 2G–I, 3B). Clinical data are summarized in Table 1. Genetic analysis revealed that the patient carried a compound heterozygous mutation of c.1033G > C (p.V345L) in exon 6 and c.3287G > T (p.C1096F) in exon 22 of the *ROBO 3* gene (NM_022370.3) (Figure 4C). The missense mutations of c.1033G > C (p.V345L) and c.3287G > T (p.C1096F) were predicted to be deleterious to protein function and structure through an online program analysis using SIFT, PolyPhen-2, MutationTaster, and CADD. The scores calculated for all mutations using the SIFT, Polyphen-2, CADD, and ACMG classifications are listed in Table 2.

**TABLE 1:** Phenotypes of individuals with HGPPS in this study.

Family	Individual ID	Age (years)	BCVA	HGP	Onset of scoliosis (years)	Nystagmus	Esotropia	“Butterfly” medulla	Uncrossed tracts
A	II:1	4	8/20	+	2	+	−	+	+
	II:2	1	NA	+	0.67	+	−	+	+
B	II:1	4	8/20	+	3	+	−	+	+
	II:2	0.17	NA	+	NA	+	−	+	+
C	II:2	11	10/20	+	6	+	−	+	+
D	II:1	16	10/20	+	6	+	+	+	+

BCVA, best-corrected visual acuity; HGP, horizontal gaze palsy; NA, not available.

**TABLE 2:** Summary of detected mutations of the *ROBO3* gene in this study.

Mutation	Protein	Exon	Domain	Type	ACMG	SIFT	CADD	Polyphen-2	Evidence levels	References
c.565dupC	p.R191Pfs*61	3	Ig-like	Frameshift	Likely pathogenic				PVS1, PM2	This study
c.1447C > T	p.R483X	9	Ig-like	Non-sense	Likely pathogenic				PVS1, PM2	This study
c.2462G > C	p.R821P	16	Fn 3	Missense	Likely pathogenic	0.097	22	−2	PM3, PM2	This study
c.1033G > C	p.V345L	6	Ig-like	Missense	VUS	0.082	28.9	3	PM2	This study
c.3287G > T	p.C1096F	22	CC	Missense	VUS	0.175	11.69	−2	PM2	This study
c.416G > T	p.G139V	2	Ig-like	Missense	Pathogenic	0	31	−3	PVS1, PS4, PM2	

**FIGURE 7:**
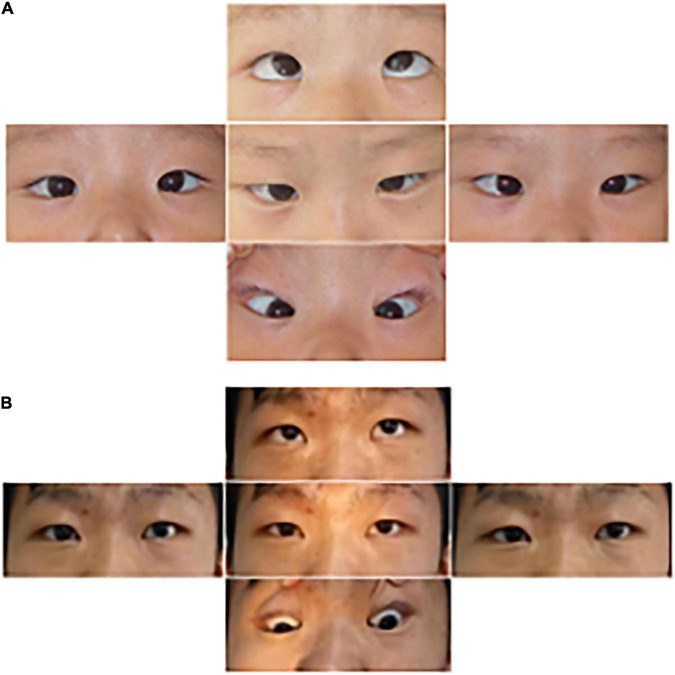
Images of the eye movements of P6. The patient shows esotropia in the primary gaze position at the age of 3 years **(A)** and residual left hypertropia after surgery **(B)**. He lacked conjugate horizontal movements but preserved vertical movements.

**FIGURE 8:**
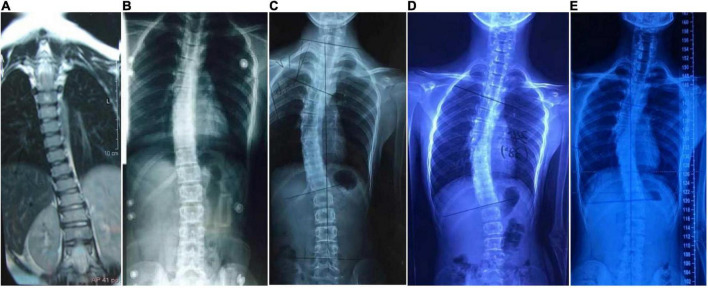
Radiology images of P6. Spinal deformation has worsened with age. **(A)**, 6 years of age; **(B)**, 9 years; **(C)**, 12 years; and **(D)**, 15 years. The scoliosis has stopped progressing after spinal orthosis **(E)**, 17 years.

Proband 6 (P6) was a 16-year-old boy (sporadic case). Medical history showed that he had undergone strabismus surgery twice before 6 years of age. The patient underwent correction of esotropia at the age of 3 years for the first time (Figure 7A) and correction of left hypertropia at the age of 5 years for the second time. HGPPS was suspected in 2012 when the patient started to present with scoliosis at 6 years of age (Figure 8A). A heterozygous variation in c.416G > T(p.G139V) in exon 2 of the *ROBO3* gene (NM_022370.3) was detected (Figure 4D). He underwent orthotic treatment for his spine with a Cobb angle of 34° at the age of 13 years. He visited our ophthalmology department for the treatment of residual left hypertropia in August 2020 as the last follow-up. Ocular examination revealed that the best visual acuity was 10/20 in each eye. He had left hypertropia with a deviation angle of approximately 15° in the primary gaze position (Figure 7B). Horizontal conjugate eye movements were completely absent, and vertical eye movement was preserved. Nystagmus was evident when the patient attempted to abduct his eyes. Radiological imaging showed that his scoliosis progressively worsened over time (Figures 1E, 8). MRI revealed a deep posteromedian cleft in the pons and a butterfly-like morphological change in the medulla oblongata (Figures 2J–L), which was different from the normal child (Figures 2M–O). DTI showed a complete ipsilateral course of the corticospinal tract without decussation at the level of the lower medulla (Figure 3C). DTI tractography of a normal child demonstrated crossed fiber bundles of the bilateral corticospinal tracts (Figures 3D,E).

## Discussion

Horizontal gaze palsy with progressive scoliosis comprises a series of rare and complex eye movement abnormalities known as congenital cranial dysinnervation disorders. Other abnormalities include congenital fibrosis of the extraocular muscles (CFEOM), Duane retraction syndrome (DRS), and Mobius syndrome. The common feature of these disorders is congenital dysplasia of the cranial nerves related to ocular movement, which is usually caused by gene mutations of *KIF21A*, *PHOXA*, and *TUBB3* in CFEOM; *CHN1* in DRS; and *ROBO3* in HGPPS (6–9).

In this study, we identified two compound heterozygous mutations, c.1447C > T (p.R483X) and c.2462G > C (p.R821P). We also identified c.1033G > C(p.V345L), c.3287G > T (p.C1096F), a homozygous indel mutation of c.565dupC (p.R191Pfs*61), and a heterozygous missense mutation in c.416G > T(p.G139V) in six patients with HGPPS. With the exception of c.416G > T(p.G139V) being reported previously as one of the compound heterozygous mutations of c.416G > T(p.G139V) and c.2108G > C(p.R703P) (10), all mutations are novel. The absence of horizontal conjugated eye movement appeared very early in our patients, with an onset time of 2–3 months after birth. Nystagmus is also a common clinical feature that could explain why patients have decreased visual acuity. However, the onset time of scoliosis varies from 8 months to 6 years after birth.

As a member of the roundabout family of transmembrane receptors, the *ROBO3* gene comprises an extracellular domain with five immunoglobulin (Ig)-like domains, one intracellular domain, three fibronectin type III extracellular motifs, and a cytoplasmic tail with three cytoplasmic signaling motifs: CC0, CC2, and CC3. It plays an important role in neurite outgrowth, growth cone guidance, and axon fasciculation, probably through interactions with SLIT proteins that play conserved roles in axon guidance and neuronal migration to regulate neurogenesis (11, 12). Four of the six pathogenic variants were identified in the Ig-like domain. Loss of the *ROBO3* gene causes a complete failure of commissural axons to cross the midline throughout the spinal cord and hindbrain in the mouse model. Human *ROBO3* mutations can lead to HGPPS, which is typically characterized by the congenital absence of horizontal gaze, progressive scoliosis, and failure of the corticospinal and somatosensory axon tracts to cross the midline in the medulla. Through imaging examination, an obvious brainstem malformation is shown as a flattened pontine tegmentum, markedly reduced facial colliculi, and reduced volume of pons and medulla, which could be related to the dysgenesis of the nucleus and nerve fiber bundles (3, 13, 14).

The neural mechanism of horizontal conjugate eye movements depends on the connections between neurons that innervate both eyes to move simultaneously in the same direction. Abducens neurons with neurons in the paramedian pontine reticular formation are regarded as the horizontal gaze center located in the tegmentum of the pons. Abducens internuclear neurons project up the contralateral medial longitudinal fasciculus to connect the motor neurons of the oculomotor nucleus, which is the anatomical basis of conjugate movement (15, 16). Mutations in the *ROBO3* gene would affect the projection of nerve fibers from one side to the other, possibly causing the absence of horizontal conjugate eye movement. Moreover, severe fibrosis of the extraocular muscles leads to restrictive horizontal conjugated eye movement. For example, patients with CFEOM caused by *TUBB3* gene mutations (E410K syndrome) may not have horizontal conjugated eye movement (4). Patients with Mobius syndrome may have horizontal conjugate eye movement defects due to dysgenesis of the six cranial nerves (17).

The etiology of progressive scoliosis is still unknown but probably involves false projections of descending fibers from the brain to the spinal cord, including the reticulospinal and corticospinal tracts, which are involved in regulating muscle tone. Our imaging data further support the idea that the maldevelopment of the hindbrain and false projections of nerve fibers are tightly associated with the absence of horizontal conjugate eye movement and progressive scoliosis. Vision plays an important role in cognitive function. Although the patient with HGPPS had visual impairment and some brain development abnormalities, no cognitive defects were found through history questioning and follow-up. In the future, functional magnetic resonance research combined with cognitive assessment will be used to evaluate the neurodevelopmental results of patients.

## Conclusion

We identified five novel mutations in the *ROBO3* gene and expanded the spectrum of *ROBO3* mutations. This can help inform future research on the molecular mechanism of HGPPS.

## Data availability statement

The datasets presented in this study can be found in online repositories. The names of the repository/repositories and accession number(s) can be found at https://www.biosino.org/node/data/public, accession number OED772543–OED772546.

## Ethics statement

The studies involving human participants were reviewed and approved by the Ethics Committee of Beijing Children’s Hospital. Written informed consent to participate in this study was provided by the participants’ legal guardian/next of kin.

## Author contributions

LH did the clinical investigation of patients and available family members with follow-up, and drafted the manuscript. JG did the imaging test and analyzed imaging data, and drafted the imaging section of the manuscript. YX, YZ, XW, and HL did the clinical investigation of patients. NL did clinical advice and critical revision of the manuscript. YP and NL supervised the conception of the study. All authors contributed to the article and approved the submitted version.
